# Microtechnologies to fuel neurobiological research with nanometer precision

**DOI:** 10.1186/1477-3155-11-11

**Published:** 2013-04-10

**Authors:** Cecilia A Brunello, Ville Jokinen, Prasanna Sakha, Hideyuki Terazono, Fumimasa Nomura, Tomoyuki Kaneko, Sari E Lauri, Sami Franssila, Claudio Rivera, Kenji Yasuda, Henri J Huttunen

**Affiliations:** 1 Neuroscience Center, University of Helsinki, P.O. Box 56, Viikinkaari 4, FI-00014, Helsinki Finland; 2 Department of Biosciences, Physiology, University of Helsinki, P.O. Box 65, Viikinkaari 1, FI-00014 Helsinki Finland; 3 Department of Materials Science and Engineering, Aalto University, School Chemical Technology, Tietotie 3, FI-02150 Espoo Finland; 4 Department of Biomedical Information, Division of Biosystems, Institute of Biomaterials and Bioengineering, Tokyo Medical and Dental University, 2-3-10 Kanda-Surugadai, Chiyoda Tokyo 101-0062 Japan; 5 On-chip cellomics project, Kanagawa Academy of Science and Technology (KAST), 3-2-1 Sakado, Takatsu Kawasaki 213-0012 Japan

**Keywords:** Microfluidics, Micropatterning, Microfabrication, On-chip technology, Axonal transport, Electrophysiology, Neurodegeneration, Neurobiology, Plasticity, Connectivity, Synaptogenesis

## Abstract

The interface between engineering and molecular life sciences has been fertile ground for advancing our understanding of complex biological systems. Engineered microstructures offer a diverse toolbox for cellular and molecular biologists to direct the placement of cells and small organisms, and to recreate biological functions in vitro: cells can be positioned and connected in a designed fashion, and connectivity and community effects of cells studied. Because of the highly polar morphology and finely compartmentalized functions of neurons, microfabricated cell culture systems and related on-chip technologies have become an important enabling platform for studying development, function and degeneration of the nervous system at the molecular and cellular level. Here we review some of the compartmentalization techniques developed so far to highlight how high-precision control of neuronal connectivity allows new approaches for studying axonal and synaptic biology.

## Review

### Introduction

Over the past decades, molecular and cellular neuroscience has taken major steps towards understanding how the nervous system develops, functions and occasionally fails in disease. With billions of neurons interconnected via trillions of synapses, the complexity of mammalian brain poses a tremendous challenge for experimental scientists. Even reduced preparations, such as cultures of dissociated primary neurons, pose significant experimental challenges due to difficulties in controlling the growth and connectivity of axons and dendrites. Chemical cues and micropatterned surfaces have long been used to control growth of neurites in cell culture conditions. Neurons adhere and grow on glass surface coated with adhesive proteins but not on native glass. Finely defined patterns of adhesive proteins can be produced by selective coating and denaturation of neurite growth-promoting proteins [1] or by microcontact printing of protein substrates on glass coverslips [2]. Axons will find their paths along the thin lines of attractive substrate while somas will be restricted to the larger coated areas.

**Figure 1:**
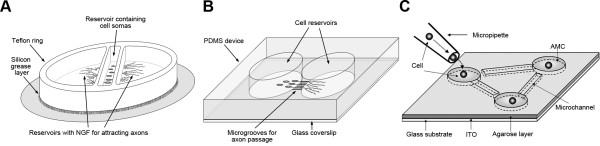
**Three techniques for isolation of axons in neuronal cell culture.** (**A**) The Campenot chamber is a Teflon ring placed on a layer of silicon grease on top of regular cell culture dish or glass coverslip. Dissociated peripheral neurons such as dorsal root ganglion cells are placed in the middle reservoir. NGF placed in the axonal chambers attracts axons to grow through the silicon grease layer while the cell somas are restricted to the middle reservoir. (**B**) Microfluidic devices are typically microfabricated from PDMS. The two cell reservoirs are connected via microgrooves (typically appr. 3–10 μm width and hight, >450 μm in length). Axons grow through the microgrooves while somas and dendrites are limited in the cell reservoirs. Many types of neurons including central neurons can be grown in microfluidic devices. (**C**) Individual neurons can be cultured to create directed neuronal networks using the low-melting agar etching technique. Glass coverslip is coated with a 50 nm-thick indium-tin iodide (ITO) layer below the agar. ITO allows highly localized photo-thermal etching of agar with a 1064-nm infrared laser beam. Based on observed initial axon growth, microchannels can be opened to connect individual neurons to form simple networks. The panel C is reproduced from [7].

Another approach to control growth and connectivity neuronal processes is compartmentalized culture systems [3]. The Campenot method used a three-compartment Teflon chamber to isolate axons from the cell bodies of peripheral neurons (Figure 1A). Axons were stimulated to grow through the sealing silicon grease layer by placing nerve growth factor (NGF) to the peripheral chambers. Since the Campenot chamber does not work well for growing central neurons, the principle of compartmentalization has been further developed and is now exceedingly utilized with a number of different materials and device geometries to provide a large number of applications to study fundamental neurobiological questions with many types of neurons (Figure 1). Compartmentalized culture systems also provide fluidic isolation that has additional benefits for chemical and genetic manipulation of neurons. This rapidly expanding field of bioengineering offers a wide array of micro-scale devices for fine control of the cellular microenvironment, growth and connectivity of neurons and their interaction with other cells of the nervous system. Here, we provide a brief introductory review of materials and techniques for manufacturing microfluidic chips and agarose microstructures, and how these microtechnologies are changing the way axonal development, connectivity, functionality and injury can be studied in the laboratory.

### Materials and manufacturing techniques

The most commonly used way to achieve micro-scale compartmentalization is through the use of microfluidic chips. Microfluidic chips contain accurately patterned features in the range of 0.1 μm - 1 mm, and thus offer highly precise spatial and temporal control of cellular microenvironment. The traditional microfabrication materials are silicon and glass, but the microfluidics community has increasingly adopted polymers such as poly(dimethylsiloxane) (PDMS) as the materials of choice [4]. Typical polymer microfabrication techniques include optical lithography, replication molding, injection molding and hot embossing. An example of an often-used polymer-based fabrication process is replica molding of PDMS [5] using masters created through epoxy polymer photoresist SU-8 photolithography, a micromanufacturing technique developed in the microelectronics industry. A recent trend is to utilize materials more familiar to biologists, such as agarose [6,7] or polystyrene [8] for microfabrication.

Other approaches to study how cells respond to the topography of the substratum (adhesion, morphology, migration), have used silicon wafers and glass slides with holes and metal decorations [9-12]. Bonding, sacrificial layer techniques, and lamination also have been used to create tunnel-shaped microstructures between two microchambers [13].

#### (a) Microfluidics: PDMS-based approaches

A commonly used approach is to utilize PDMS microfluidic chips sealed on top of glass. The wide adoption of PDMS stems mainly from ease of fabricating even large batches of chips through casting. The casting process (other than the master fabrication) also does not require any dedicated equipment, and can be performed anywhere. In addition, PDMS is a biocompatible and optically transparent polymer that forms reversible watertight seals when placed on smooth surfaces due to its elasticity [4]. PDMS can be permanently bonded with glass or other PDMS layers by first activating both surfaces with plasma (most commonly oxygen plasma). The plasma treatment also turns PDMS hydrophilic, albeit only temporarily [14]. Other possibilities for PDMS-glass bonding include adhesive bonding with a polymeric adhesive (a glue) or a single molecular layer thick surface adhesive [15]. A typical architecture of microfluidic chips used in neurobiological applications consists of axon isolation microgrooves that are connected to two or more larger reservoirs that house the cell populations either directly (Figure 1B) [16] or through larger microfluidic channels [5]. The dimensions of the microgrooves need to be chosen so that they are large enough for the axons to enter, but too small for the cell somas to fit in. Typically dimensions in the range of 2 – 10 μm are used for this purpose. The length of the microgrooves can be used to control whether or not dendrites are also able to traverse between compartments. Compared to axons that can grow many millimeters (and more), dendrites typically penetrate only 100 – 300 μm into the microgrooves [5,16,17].

#### (b) Micropatterning techniques: Agar-microstructure technology

Significant advances have been made in developing analytical methods to monitor neuronal activity on a single cell level (fluorescence imaging, voltammetry, ion-selective electrodes, microelectrode arrays, combination of separation techniques with mass spectrometry) [18-20]. To meet the spatial resolution of those single cell level-monitoring technologies, micropatterning techniques for controlling of adequate spatial arrangements of neurons and neurites have been developed and applied [21,22]. While most of micropatterning techniques such as microcontact printing and microetching-based fabrication techniques are suitable for controlling the populations of dissociated neurons with randomly arranged network patterns, those conventional micropatterning techniques can just control the orientation of spatial arrangements of neurites in pre-fabricated (ready-made) micropatterns, and, in principle, cannot control the directions of neurites’ elongation and connections. To overcome those problems, agar-microetching technique has been developed to fully control of spatial arrangements of single neurons and the direction of their connectivity by flexible stepwise-fabrication of additional microstructures (Figure 1C) [6,7,23]. This technique provides a constructive approach for spatial direction control and neuronal network formation during cultivation.

**Figure 2:**
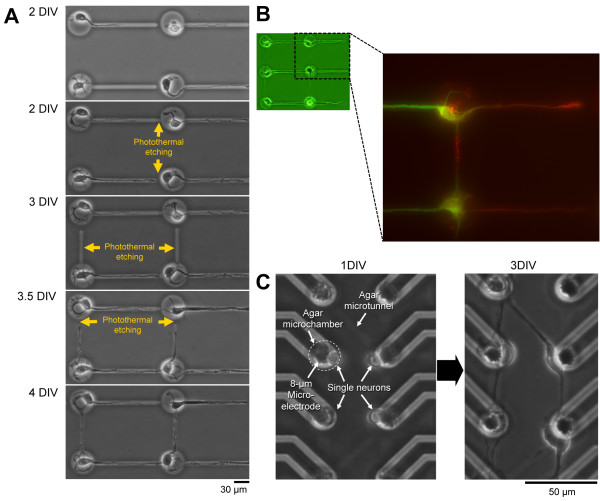
**Topographic control of axon/dendrite connections by stepwise formation of neuronal network pattern using photothermal etching.** By applying stepwise photo-thermal etching to agar microchambers during cultivation, the direction of synaptic connectivity in a living neuronal network can be controlled. This allows the tunnels in which axons were elongated to be flexibly extended by melting the narrow micrometer-order grooves (microchannels) in steps through photo-thermal etching where a portion of the agar layer is melted with infrared laser beam. (**A**) Phase-contrast images of single hippocampal neurons during the stepwise etching procedure. When cultivation started, single cells were placed into the agar microchambers and cultivated for 2 days in vitro (DIV). The first single neurites that elongate from the cells into the microchannels are typically axons, whereas the second and latter neurites are typically dendrites. When the elongations of neurites are sufficiently stable, additional photo-thermal etching steps can be applied to connect two adjacent agar microchambers. (**B**) Fluorescent staining of neurites with axon- and dendrite-specific markers at DIV 5 (red, synapsin I for axons; green, MAP2 for dendrites). (**C**) A combination of microelectrode and agar microchambers for an individual-cell-based neural network patterning and electrophysiological recording. 8-μm microelectrode-size agar microchambers and 2-μm microtunnels were arranged on the MEA chip to control the positions of neurons and their connections.

Agarose microstructures can be photothermally etched by area-specific melting of agar microchambers by spot heating using a focused laser beam of 1480 nm (which is absorbed by water and agar gel), and of a thin layer made of a light-absorbing material such as chromium with a laser beam of 1064 nm (since water and agar itself have little absorbance at 1064 nm) [24]. For phase-contrast microscopy and μm-scale photo-thermal etching, three different wavelengths (visible light for observation, and 1480-nm/1064-nm infrared lasers for spot heating) were used simultaneously to observe the positions of the agar chip surface and to melt a portion of the agar in the area being heated. Using this non-contact etching, microstructures such as holes and tunnels can be created within a matter of minutes (Figure 2).

### Neurobiological applications: focus on axons

By offering many significant methodological advantages, microfluidic techniques are reshaping the way axonal cell biology is studied in vitro. As compartmentalized microfluidic devices are based on geometric guidance of axon growth via engineered microgrooves, they provide a high level of control of connectivity and architecture of the neuronal network. This provides significant benefits for biochemical (e.g. isolation of axon-specific mRNA and protein species), cell biological (e.g. kinetic studies of axonal transport of vesicular and organelle cargo) and electrophysiological studies (e.g. recording the functional consequences of axon-specific manipulations) [5,16,25-27]. Specific chips using microfluidic isolation have been developed for production of large quantities of material for proteomic and RNA analyses of axonal composition [2].

#### (a) Axonal growth, myelination and synaptogenesis

The ability to image and quantify growth properties of axons and dendrites (growth rate, length, fasciculation, retraction etc.) in microfluidic devices is superior over more traditional methods [17,28]. Moreover, microfluidic devices are well suited for studying interactions of neurons with their target cells or with glial cells. Neuron-glia co-culture devices provide means for studying CNS axon-glia interaction, such as neuron-oligodendrocyte signaling during myelination [29]. In a microfluidic neuron-oligodendrocyte coculture device with cell-reservoir immersed electrodes, constant electrical stimulation was shown to increase myelination of axonal segments [30]. Acetylcholine receptor clustering during neuromuscular synaptogenesis was studied using differentiated myotubes and laminar flow of agrin in microfluidic devices [31]. Combination of microfluidic chips with actuators, such as pneumatically or hydraulically controlled valves, for fine control of neuron-to-neuron connectivity provides another interesting approach for studying synaptogenesis in vitro [32].

#### (b) Axonal transport

The functionality of the axon and the presynaptic terminal is critically dependent on axonal trafficking of materials (protein, mRNA, lipids) and organelles such as mitochondria. Moreover, retrograde transport of target-derived signals, particularly neurotrophic proteins, is essential for neuronal survival and plasticity [33]. Recent work has utilized microfluidic devices for single-molecule imaging of retrograde axonal transport of NGF [34] and dendrite-to-nucleus signaling of brain-derived neurotrophic factor (BDNF) [35]. Defects in axonal transport play an important role in pathogenesis of neurodegenerative diseases [36]. β-amyloid peptide (Aβ) and Tau are central pathophysiological molecules in Alzheimer’s disease. Using microfluidic devices, it has been shown that both Aβ [37] and Tau [38] impair axonal transport of mitochondria, and that Aβ also impairs retrograde axonal transport of BDNF and its receptor TrkB [39]. Interestingly, misfolding proteins that are characteristic for many neurodegenerative diseases appear to propagate between neurons in a prion-like fashion. Microfluidic devices provide an effective means to study neuron-to-neuron propagation mechanisms in vitro, as shown by a recent study addressing anterograde propagation of α-synuclein fibrils [40].

#### (c) Axonal injury and degeneration

One of the main advantages of spatial segregation of axons is the possibility to investigate specific mechanisms of CNS axonal injury and degeneration, as well as possible regeneration [41]. Since axonal injury and degeneration are closely associated with pathophysiology of traumatic brain injury and many neurodegenerative diseases, a number of approaches have been developed to study axonal injury in vitro. Initial attempts to perform axotomy used mechanical cut [42], but the lack of reproducibility and poor spatial control underlined the need of new techniques. Microfluidic axon isolation devices allowed better control on axonal growth and injury (using vacuum aspiration or mechanical transection of axons) [5,26]. Microfluidic devices have been used in combination with many types of experimental axonal injuries. Focal laser irradiation, using either femtosecond laser [43] or less harmful pico- and nanosecond laser [44], allows precise and localized axonal damage. In three-compartmental microfluidic devices, which have a cleft in between the somatic and the distal axonal chambers, injury has been performed by flux of detergent [45] and by valve-based micro-compression [46]. Other multi-compartment chips, designed to study axon-glia interaction [29], provide information on migration and functional interaction of glial cells with damaged axons after injury [47,48].

### Applications for studying synaptic biology

Because of their central role in biological computation and storage of information, synapses are of special interest in neurobiology. A synapse represents a very compact subcellular compartment with diameters less than 1 μm. Understanding the detailed molecular events that regulate presynaptic function requires high-resolution methods that provide quantitative information combined with molecular specificity. In recent years, numerous technological advances, including optogenetic methods and live-cell imaging, have facilitated probing the physiology of individual synapses. Combination of these techniques with microfluidic culture platforms offers a powerful way to study synaptic biology.

#### (a) Imaging based techniques

Fluorescent organic dyes are widely used as reporters of dynamic changes in various cellular processes. Because calcium ions control key functions in all types of neurons, imaging dynamic local changes in intracellular calcium concentration has become an important experimental readout in neurobiology [49]. Pioneering studies show that combination of calcium imaging with microfluidic applications, such as controlling neuronal connectivity and local perfusion in vitro [17] or immobilization of a whole organism [50], can provide powerful ways to image dynamics of neuronal processes. Moreover, fluorescent reporter proteins combined with microfluidic devices have been used to study how synaptic transmission is coupled to local changes in dendritic protein synthesis, a prerequisite of many forms of synaptic plasticity [25], and for quantitative analysis of presynaptic receptor localization and axon-dendrite contact formation [51].

#### (b) Single-cell recording

Electrophysiological methods and in particular, the current clamp and voltage clamp techniques, provide the best temporal resolution for functional analysis of excitable cells. Intracellular recording methods typically target the postsynaptic cell, and allow high precision analysis of somatodendritic function. PDMS-based microfluidic technologies have been developed for high-throughput electrophysiological studies [52,53]. In these applications, precisely engineered microfluidic junctions in a chip substitute the conventionally used glass electrode, allowing simultaneous patch clamp recordings to be made from a large number of cultured cells. These techniques have been combined to fast precisely controlled application of pharmacological substances, such as neurotransmitters. For example, a combination of microcontact-printed microstructured chips (with strict geometrical control over the network architecture) aligned with a microfluidic device for accurate application of chemical stimulants was used for whole-cell patch clamp recording in reconstructed networks of cortical neurons [54]. Moreover, loose patch clamp technique built on a PDMS device for spatial isolation of soma and neurites was developed to detect multi-unit activity of neurons [55].

Apart from the high-throughput systems, microfluidic chambers allowing compartment-specific manipulation provide valuable tools to study neuronal and in particular, axonal function. Geometrical limitations of PDMS-based culture platforms have made it challenging to combine conventional microelectrode-based patch clamp recording with microfluidic control of neuronal connectivity. Recently, a simple modular, three-layer PDMS chamber was developed for controlling neuronal connectivity and asymmetric viral transduction while also allowing whole cell patch clamp recording [16]. Together with optogenetic stimulation of the presynaptic neurons, this system allows effortless recording of single synapse activity, with the possibility for selective experimental manipulation of fluidically isolated neuron populations.

#### (c) Multielectrode arrays

Non-invasive extracellular measurement using a multi-electrode array (MEA) has been widely used in neuroscience for the past two decades [56-61], and has proven to be an effective long-term electrophysiological measurement technique for neural cells. Integration of microfluidic control of neuronal connectivity with non-invasive extracellular measurement using MEA provides a new avenue for experimentation [23,62]. In addition to the simultaneous multisite recording capability of this approach, it also gives the possibility to specifically stimulate different neuronal compartments (dendrites, soma and axons). This feature provides a powerful method to elucidate compartment specific mechanisms and also offers interesting approaches for drug screening [63]. Using a combination of microfluidic chip with MEA for stimulation and recording, Takeuchi et al. cultured superior cervical ganglia neurons and ventricular myocytes separately on microfluidic chips and showed that the MEA-controlled firing rate of cervical neurons could modulate the beat rate of cardiomyocytes [64].

Planar electrodes are suitable for monitoring fluctuation in the extracellular field potential produced by small groups of neurons when it is difficult to record and stimulate single neurons. In addition to the microwell-based approach of physically restraining connectivity of neurons with planar electrodes [23,65], an interesting line of development exploits the ability of microchannels to conduct electrical signals to achieve single cell patch recording. PDMS electrodes with modified surface chemistry and a subterranean microfluidic channel are capable of forming high-resistance seals with cell membranes, and have been used as a basis for planar patch clamp systems [66]. Recently, this approach was combined with MEA to create a system that allows simultaneous probing of individual neurons and high-resolution MEA recording at multiple sites in synaptically connected neuronal networks [67]. Further miniaturization of channel arrays will allow more precise control of the extracellular space as well as membrane potential at the sub-compartment level [68]. Moreover, advances in microfabrication technologies have enabled the creation of electrode arrays that can be used to simultaneously observe the firing of multiple cells, but the problems of contamination and cells escaping from the position of each electrode remain and often occur in long-term cultivation.

#### (d) Directed neuronal networks

In neuroscience, one of the main interests is how epigenetic information is processed and recorded as plasticity within a network pattern, and how changes in the network patterns relate to plasticity. Thus, for many years, neurobiologists have investigated single-cell-based neuronal network cultivation and examined the firing patterns of single neurons through the fabrication of cultivation substrates using microprinting techniques [69-71], patterning on silicon-oxide substrates [72], and three-dimensional structures made using photolithography [73]. Although these conventional microfabrication techniques provide structures with fine spatial resolution, effective approaches for studying epigenetic information are still being sought. With conventional techniques, it is still hard to make flexible microstructures with simple steps or to change their shape during cultivation since the shape is usually unpredictable and only defined during cultivation.

Using the photo-thermal etching method, microstructures can be formed within the agar layer on the chip by melting a portion of the agar layer at the spot of a focused infrared laser beam as shown in Figure 2A [7,23]. This method can be applied even during cultivation, so the network pattern of nerve cells can be changed during cultivation by adding microchannels between two adjacent microchambers in a step-by-step fashion. This helps us to understand the meaning of the spatial pattern of a neuronal network by comparing the changes in signals before and after the network shape is changed. For example, an agar microchamber placed on top of an MEA can be used to record the long-term electronic properties of topographically controlled neuronal networks with precise fixation of cell positions and flexible network pattern rearrangement through photo-thermal etching of the agar layer.

An MEA system with an agar microchamber to control the topography of network patterns of neuronal networks in single cell level has been accomplished (Figure 2C) [74]. The advantages of this combination of MEA-agar microchamber system are that it allows multiple cells firing simultaneously to be recorded for weeks without contamination, and that it allows cell positions, numbers, and their connections for cultivation to be controlled using agar microchambers with microchannels fabricated by photothermal etching where a portion of the agar layer is melted with a 1480-nm infrared laser beam. This method allows individual-cell-based neural network patterning and electrophysiological recording without cells escaping from single cell-size (8 μm) electrode positions in the single-microelectrode-size agar microchamber array.

Asymmetric microchannels in PDMS chips provide another way for creating unidirectional axon connectivity. Peyrin et al. recently described reconstruction of oriented cortico-striatal neuronal networks in “axon diode” microfluidic chambers composed of funnel-shaped microchannels [75]. The axon diode chip provides an important development, as it is a batch-producible device for creation of highly ordered neuron connection architectures in vitro.

### Applications for studying whole organisms

Handling, sorting and analysis of small model organisms widely used in biology, Caenorhabditis elegans and Drosophila melanogaster, have been facilitated by developing special microfluidic devices [76]. Precise anesthesia-free positioning and immobilization of small model organisms for in vivo microscopy also greatly benefits from microfluidic devices. For example, neurobiologists studying small model organisms have found microfluidic devices useful for imaging neuronal transport of GFP-tagged cargos in living C. elegans and Drosophila larvae [77] and for studying synaptic transmission in neuromuscular junction in C. elegans [78]. Also, microfluidic device-assisted live animal studies of axonal injury have been performed in C. elegans [79] and in Drosophila larvae [50].

## Conclusions and future prospects

Microfluidics has the potential to revolutionize cell biology. In particular, neurobiologists have found microfluidics capable of providing solutions to many methodological challenges. The number of device designs and applications is increasing rapidly, and microfluidic techniques are likely to be adopted widely by the neuroscience community in the near future. Integrated designs using combination of microfluidic chips with MEA, optogenetics and various types of biosensors (real-time detection of neurotransmitters and neuropeptides etc.) is expected to take these emerging on-chip technologies to new heights. Moreover, microfluidic devices are increasingly used with brain slices and could also provide great opportunities as scaffolds to develop implantable neural interfaces. Finally, utilization of the third growth dimension will provide another future direction to this new field of *neurofluidics*.

## Abbreviations

Aβ: β-amyloid peptide; BDNF: Brain-derived neurotrophic factor; CNS: Central nervous system; GFP: Green fluorescent protein; MEA: Multielectrode array; NGF: Nerve growth factor; PDMS: Poly(dimethylsiloxane); PLL: Poly-L-lysine.

## Competing interests

H.J.H is a cofounder and a shareholder of Hermo Pharma Ltd. Other authors declare no conflict of interest.

## Authors’ contributions

CAB and PS performed literature review and contributed in writing the first draft of the manuscript. VJ and SF wrote the section on PDMS-related materials and manufacturing techniques. HT, FN, TK and KY wrote the sections on agar-microstructure technology and directed neuronal networks. SEL and CR performed literature review and contributed in writing the electrophysiological parts of the review. KY and HJH designed, edited and revised the final manuscript. All authors read and approved the final manuscript.
